# Appropriateness of Therapeutic Antibiotic Prescriptions by Lebanese Dentists in the Management of Acute Endodontic Abscesses

**DOI:** 10.7759/cureus.7327

**Published:** 2020-03-19

**Authors:** Ghada Al Asmar Ramli, Jacques E Mokhbat, Dominique Cochelard, Mohamed Lemdani, Ahmed Haddadi, Fouad Ayoub

**Affiliations:** 1 Orthodontics, Faculty of Dental Medicine, Lebanese University, Beirut, LBN; 2 Infectious Disease, School of Medicine, Lebanese American University, Beirut, LBN; 3 Epidemiology and Public Health, Laboratoire Biomathematiques, Lille 2 University, Lille, FRA; 4 Epidemiology and Public Health, Laboratoire Biomathematiques, Lille University, Lille, FRA; 5 Public Health: Epidemiology and Quality of Care, Lille 2 University, Lille, FRA; 6 Forensic Odontology and Human Identification, Faculty of Dental Medicine, Lebanese University, Beirut, LBN

**Keywords:** endodontics, dentistry, antibacterial agents, drug resistance

## Abstract

The misuse of antibiotics in dentistry is a serious concern especially in regards to the emergence of antibiotic resistance. The objective of the study was to evaluate the appropriateness of antibiotics prescriptions by Lebanese dentists to patients with endodontic abscesses and their compliance with the guidelines of the American Association of Endodontists (AAE) and the European Society of Endodontology (ESE). The treating dentists recorded clinical information from 127 patients diagnosed with acute or chronic endodontic abscess. The information also included the type of antibiotic prescribed, dosage, and duration of the prescription. Prescriptions were not given to 14/20 patients with an acute endodontic abscess despite the presence of an indication to prescribe. All the prescriptions given to patients with an acute endodontic abscess were inappropriate according to the ESE and AAE guidelines. Antibiotics were also prescribed unnecessarily to 17/42 patients with a chronic endodontic abscess. This study concluded that antibiotics prescriptions by Lebanese dentists for the management of endodontic abscesses were inappropriate. Penicillin V potassium (VK) was not available in Lebanon when the study was conducted. Only broad-spectrum antibiotics were prescribed. This finding raises concerns about the emergence of antibiotic resistance.

## Introduction

Most endodontic infections can be managed by drainage, endodontic treatment, or extraction [1-2]. However, in specific situations, these local measures may not be adequate and the prescription of antibiotics may be recommended [3-5]. The European Society of Endodontology (ESE) and the American Association of Endodontists (AAE) proposed guidelines regarding the indications to prescribe antibiotics, the type of antibiotic, and the prescription regimen in the presence of oro-facial infections of endodontic origin as a therapeutic measure [3-4].

Several studies have demonstrated that dentists from different jurisdictions do not comply with the recommended guidelines [6-13]. The non-compliance included the indication to prescribe, the type of antibiotic, and the regimen of the prescription. Moreover, several articles have raised concerns about the relationship between the inappropriate use of antibiotics and the emergence of antibiotic resistance [14-16].

There are no national guidelines in Lebanon for the therapeutic prescription of antibiotics in dentistry. One study suggested that Lebanese dentists did not follow international guidelines [17]. More recently, another study raised a similar concern despite the fact that Lebanese dentists were, in general, aware of the relationship between the emergence of bacterial resistance and antibiotic misuse [18].

The objective of the present study was to evaluate the compliance of Lebanese dentists with the AAE and ESE guidelines for the prescription of therapeutic antibiotics for dental infections of endodontic origin.

## Materials and methods

This study was approved by the Committee of Ethics, Faculty of Dentistry, Lebanese University, and was conducted over nine months. It involved patients from the dental clinics at the Faculty of Dentistry, Lebanese University, and 24 private dental clinics located in Beirut and its outskirts. Eligible patients met the following criteria: men or women aged 16 and above, clinically diagnosed with an acute endodontic abscess (AEA) or a chronic endodontic abscess (CEA) [19]. Patients who needed hospitalization were not included in the study. All dentists involved with these clinics followed a refresher course on the diagnosis of endodontic disease with emphasis on acute and chronic endodontic abscesses.

The dentists were asked to record the information presented in Table 1 when a patient presented with an AEA or a CEA.

**Table 1:** Clinical parameters collected for each patient

Diagnosis of acute or chronic apical abscess
Fatigue
Fever
Trismus
Lymphadenopathy
Difficulty in swallowing
Blurry vision
Medically compromised patient
Progressive infection (rapid onset 24h, progressive swelling, osteomyelitis, cellulitis)
Persisting infection
Extraction
Incision and drainage, intracanal drainage
Endodontic treatment
Antibiotic prescription (type, dosage/loading dose, duration)

At the end of the study period, information was collected. The information for each patient to whom antibiotics were prescribed was assessed for compliance with the ESE and AAE guidelines with regards to the indication to prescribe antibiotics and the antibiotic prescription (type, dosage, and duration).

## Results

One-hundred twenty-seven patients were diagnosed with either an acute (n=85) or a chronic (n=42) endodontic abscess. The information collected from the different clinics was complete for all the patients.

Seventeen patients with a chronic endodontic abscess were prescribed therapeutic antibiotics unnecessarily and contrary to the ESE and AAE guidelines.

Antibiotics were not prescribed to 20/85 patients with an AEA. The remaining 65/85 patients received a prescription. The antibiotics were inappropriately prescribed to 62/65 patients with an AEA according to the ESE and AAE guidelines. The inappropriateness involved the indication to prescribe, the prescribed antibiotic, the dosage, and the duration of the prescription. Prescriptions non-compliant with the ESE guidelines alone were given to 3/65 patients (Table 2). None of the prescriptions was non-compliant with the AAE guidelines alone.

**Table 2:** Patients with AEA and with an antibiotic prescription: non-compliance with ESE and AAE guidelines. *AEA: Acute endodontic abscess; **ESE: European Society of Endodontology; ^+^AAE: American Association of Endodontics

Total number of patients with AEA* and with a prescription	Total number of patients with AEA* and with a prescription, and without compliance with ESE** or AAE^+^	Total number of patients with AEA* and with a prescription, and without compliance with ESE** and AAE^+^	Total number of patients with AEA* and with a prescription, and without compliance with ESE**	Total number of patients with AEA* and with a prescription, and without compliance with AAE^+^
65	65	62	3	0

Antibiotics were not prescribed to 20/85 patients with an AEA. Six of these patients (6/20) were appropriately managed according to the AAE and ESE recommendations: those patients did not need a prescription. Fourteen patients (14/20) were not well managed according to the ESE/AAE guidelines: they were not given a prescription despite the presence of an indication to prescribe. For 6/14 patients, the absence of the prescription was not compliant with the ESE and AAE guidelines; i.e., a prescription should have been given to these patients. Non-compliance with the ESE guidelines alone involved an additional eight patients (Table 3).

**Table 3:** Patients with AEA and without an antibiotic prescription: non-compliance related to indication. *AEA: Acute endodontic abscess; **ESE: European Society of Endodontology; +AAE: American Association of Endodontics

Total number of patients with AEA* and without a prescription	Total number of patients with AEA* and without a prescription, and without compliance with ESE** or AAE^+^	Total number of patients with AEA* and without a prescription, and without compliance with ESE** and AAE^+^	Total number of patients with AEA* and without a prescription, and without compliance with ESE**	Total number of patients with AEA* and without a prescription, and without compliance with AAE^+^
20	14	6	14	6

## Discussion

The clinical situations presented in Table 1 were used by the ESE and AAE to determine the need to prescribe antibiotics [3-4]. They were included in the information collection in the present study because they allow the determination of the degree of compliance with ESE and AAE guidelines.

To the knowledge of the authors, this was the first prospective observational study to evaluate the prescribing patterns of antibiotics by Lebanese dentists in the presence of an endodontic abscess and the compliance of the prescriptions with the ESE and AAE guidelines.

During a period of nine months, a total of 127 patients presented with apical disease, chronic (42 patients) or acute (85 patients). Prescriptions were given to 85 patients (63.78%); 65 and 20 patients with an AEA or CEA, respectively. Antibiotics were not given to 20 and 25 patients with an AEA or CEA, respectively.

Non-compliance of prescriptions with the ESE and AAE guidelines occurred with 65 patients with an acute endodontic abscess and was related to the indication to prescribe (n=46) and the adequacy of the prescription, including antibiotic molecule, dosage, and duration (n=51). It was noteworthy that Penicillin VK, a narrow-spectrum antibiotic, was not prescribed to any patient. Penicillin VK was recommended by the AAE and ESE as the antibiotic of choice for the management of endodontic infections [3-4]. It appeared that this antibiotic was not available in Lebanon at the time of the study. Dentists instead prescribed a broad-spectrum antibiotic for every patient included in the present study. This finding raised serious concerns about the emergence of resistant bacteria strains associated with the use of broad-spectrum molecules [20-21]. The World Health Organization stressed that antimicrobial resistance to antibiotics is a global health concern [22]. The other elements of non-compliance such as the indication to prescribe and the duration and dosage of the prescription would also be implicated in the emergence of antibiotic resistance [21].

Antibiotics were inappropriately prescribed to 62 patients according to the AAE guidelines. For these same patients, the prescriptions were not compliant with the ESE recommendations. An additional three patients were given prescriptions non-compliant only with the ESE guidelines. Those three patients were medically compromised and presented with a diffuse swelling, which was an indication to prescribe according to the ESE guidelines. However, the patients were prescribed amoxicillin instead of penicillin VK as recommended by the ESE. The prescriptions for these patients were compliant with the AAE guidelines according to which similar patients should receive amoxicillin. Therefore, the difference between the number of prescriptions non-compliant (n=3) with the ESE (n=65) and the AAE (n=62) was related to differences in the guidelines involving the prescription (n=3).

Non-compliance did not only involve prescriptions given to patients presenting with an acute endodontic abscess. Non-compliance was also found with eight patients who were not prescribed antibiotics despite the presence of an indication to prescribe. Five patients would receive an antibiotic prescription if the AAE guidelines were followed. The same five patients would be given a prescription according to the ESE guidelines. An additional three patients should have received a prescription following the ESE recommendations. The difference between the number of patients who were mismanaged by not prescribing antibiotics (n=3) according to the ESE (n=8) and the AAE (n=5) confirmed the presence of differences between the ESE and the AAE with regards to the indication to prescribe antibiotics to patients with an AEA. All of these three patients were medically compromised and presented with localized edema, which is an indication to prescribe antibiotics, in addition to performing a root canal treatment according to the ESE but not the AAE, which recommends the initiation of a root canal procedure to control the infection in the presence of localized edema, instead of prescribing antibiotics [4].

It was also noteworthy that dentists prescribed antibiotics unnecessarily, and contrary to the ESE and AAE guidelines, to 17/42 patients presenting with a CEA. It has been well-established that therapeutic antibiotics should not be given in these situations [3-4]. Considering that all the 17 prescriptions, to patients who will not benefit from them, consisted of a broad-spectrum antibiotic, this finding raised the authors’ concerns regarding the occurrence of pseudomembranous colitis and antibiotic resistance, which are more likely to develop with this type of antibiotic [20-21,23].

## Conclusions

In agreement with other studies, the present study demonstrated that dentists, in general, prescribed antibiotics inappropriately in the presence of dental infection. More specifically, this study confirmed the impression that Lebanese dentists have poor antibiotics-prescribing patterns in the presence of dental infection. The findings were an indication of the lack of knowledge of the dentists. This aspect should be investigated especially with regards to the curriculum taught in dental schools in Lebanon. At a national level, the unavailability of narrow-spectrum antibiotics in Lebanon should be alarming.
